# Ecology or Health—How to Successfully Promote Palm Oil Free Products: A Comparison between Spain and Poland

**DOI:** 10.3390/foods10102380

**Published:** 2021-10-08

**Authors:** Dominika Adamczyk, Dominika Maison

**Affiliations:** Faculty of Psychology, University of Warsaw, Stawki 5/7, 00-183 Warsaw, Poland; dominika.maison@psych.uw.edu.pl

**Keywords:** palm oil, palm oil substitutes, consumer decisions, chocolate bread spread, ecological attitudes

## Abstract

Palm oil, widely used in the food industry, is causing some concern due to its negative impact on the environment and human health. The goal of the conducted research was to answer the question of what would be a better strategy for the marketing communication of palm oil substitutes, its health benefits or its environmental friendliness? This article presents a research project exploring the potential of chocolate bread spread based on a saturated fat and palm oil substitute. The research was conducted on two samples of Spanish (*n* = 675) and Polish (*n* = 661) bread spread consumers. In the experimental study, consumers were presented with a description of a new chocolate spread entering the market, with references to (a) its health benefits or (b) its environmental benefits resulting from the absence of palm oil in the product. The results showed that ecology references in food-related marketing communication in Spain have a stronger influence on the consumer decision-making process than health references. In Poland, the effect of communication was moderated by an evaluation of a person’s eating style and the individual level of eco-friendly behavior of the consumer.

## 1. Introduction

Palm oil, widely used in the food industry for many years, is causing some concern due to its negative impact on the human body (high saturated fat content and, most importantly, toxic substances resulting from the overheating of palm oil during the refining process [1,2]), the state of the environment (e.g., devastation of tropical forests and greenhouse gas emissions as a result of palm oil production), and the social issues concerning the people involved in its production (e.g., land grabbing and violation of labor rights).

In the face of an increasing consumer awareness of the food they consume, several issues were raised about the need to find a solution to the palm oil problem, even up to consumer boycotts of companies using it (e.g., Nutella [3]). Studies on consumer attitudes towards palm oil indicate that—given the many problems arising from the consumption and production of this type of oil—health and environmental issues are the issues that consumers are most concerned about [4]. Social issues like the exploitation of employees and pollution of local areas are less important for consumers. As an industry answer to consumers’ concern about palm oil, some brands have put information stating “palm oil-free” on the packaging of their products, while others have decided to use palm oil from safe and sustainable plantations [5]. However, as the research shows, the concept of sustainable palm oil is not entirely convincing to consumers [5]. Companies are, therefore, endeavoring to somehow replace this product with other fats, but products based on palm oil substitutes are often not as smooth as palm oil and do not give as much pleasure to the palate and senses. In this context, the question arises as how to make palm oil alternatives attractive to the consumer, encouraging them to switch from their current brand and try a new product?

On the one hand, the subject of ecology is rapidly growing in popularity and has been communicated through the actions of many companies for quite some time now. On the other hand, research seems to suggest that ecology-related arguments are not necessarily convincing for consumers and, despite the declared interest, does not translate into actual behavior [6]. Therefore, researchers are trying to explain what makes people behave in an environmentally friendly way [7]. Such studies mainly address demographic issues such as age, gender, level of education, and income [8]. Other studies highlight the importance of personality traits like openness or altruism [9]. Yet other researchers are pointing out that a strong predictor of environmentally friendly behavior can be pro-ecological attitudes [10] or knowledge about ecology [11]. The level of environmental care or of a pro-environmental attitude is a predictor of such ecological behavior as ecological consumer decisions in general or buying organic food [12]. The presented studies are in line with the results of research dating back to the 1970s, which state that educating the public about the harmful effects of certain activities on the environment should result in an increase in pro-ecological attitudes and, as a result, in a change in behavior to become more environmentally friendly. However, nowadays, such direct relationships between knowledge, attitudes, and behaviors are called into question [8]. Some studies show that those with pro-ecological attitudes do not always translate their worldview into behavior or consumer decisions [6].

Many studies have shown that attitudes towards ecology and the environment are largely influenced by culture, which is why people in different countries have divergent approaches to ecology. Researchers draw attention to the fact that inhabitants of different countries understand the value of ecology differently [13]. As the Oliver and Lee study shows, the manner in which consumers react to products that improve the environment or are environmentally friendly is also culture dependent [14]. There are even certain differences in approach to this topic on the level of countries from the same continent, such as Europe, for instance, where studies have shown variance in attitudes to more general sustainable behavior between the inhabitants of European countries [15].

The above studies show that the effectiveness of arguments related to the environmental benefits of products depends on many individual factors. Moreover, despite the declared interest, they do not translate into actual behavior. This would imply that health references should be more effective from a marketing point of view. The problem is, however, that health arguments relating to food products have a negative influence on the perceived taste of a product.

In the context of palm oil, taste plays a key role as, on the one hand, the addition of palm oil makes the product smooth and, therefore, tasty. Consequently, its absence potentially makes the product less tasty. Moreover, references to the health benefits of giving up palm oil can lead to a “healthy = not tasty” effect, thus remaining unconvincing for consumers. The studies by Sodano, Riverso, and Scafuto [4] regarding the factors influencing the renunciation of palm oil point to the equal importance of health and environmental effects. However, the authors draw attention to the fact that the effect of health damage is significantly influenced by age. They explain this result by the fact that health is of particular importance for elderly people. What is more, according to the authors, the media communicating the harmfulness of palm oil refer largely to its health effects, which may also affect the consumer perception of the product.

Getting to the bottom of consumer responses to messages relating to different types of product values may help companies make better managerial decisions concerning the positioning and communication of a product with palm oil substitutes. Are references to fostering the natural environment in brand communication effective and worthwhile from the consumer’s perspective? It is likely that consumers react to the ecological and health attributes of products in different ways? Their reactions may depend on numerous demographic, psychological, and cultural traits. The study comparing the approach of Spanish and Polish consumers to chocolate bread spread containing palm-oil substitutes, involving a self-assessment of their eating style, will be discussed in detail in this article.

### The Aim of the Article and Hypotheses

The aim of this article is to examine the perception of palm oil-free products in relation to different arguments. Based on the results from previous research on the topic, we decided to focus exclusively on health and ecological concerns. We wanted to examine whether there are any differences in the reception of messages concerning the health and ecological values of products containing palm oil substitutes. As research shows, the approach to ecology depends on individual characteristics such as knowledge, attitudes, and beliefs about nutrition, hence, we also wanted to ascertain whether an assessment of one’s own eating style influences the way given arguments are perceived. In addition, we wanted to compare Spanish and Polish consumers in terms of their reactions to arguments concerning a new palm oil-free product.

The following hypotheses were made:

**Hypothesis** **1** **(H1).** *A food product labelled as palm oil-free for health and ecological benefits will be better evaluated than a food product without the specified argument. However, a product referring to health reasons will be rated better than a product referring to ecology.*

**Hypothesis** **2** **(H2).** *Communication referring to health benefits will have a greater impact on the positive assessment of a product in people who are more health-oriented, and communication referring to ecology in more ecology-oriented consumers.*

Although the research described above does not concern the attitude of Spanish and Polish citizens towards ecology in the context of nutrition, nevertheless, it allows some differences to be identified in the attitude of Spaniards and Poles on the subject of sustainability in general. While research to date does not allow for specific hypotheses, because ecology is a relatively new topic in Eastern European countries, it was assumed that, in terms of the general awareness of their citizens, the residents of Poland will react less positively to ecology arguments.

We also wanted to ascertain whether demographic variables will be related to the approach to palm oil free products. However, with regard to demography, due to its exploratory nature, no hypotheses were posited.

## 2. Method

### 2.1. Sample

The study was conducted on the following online platforms: Ariadna (Polish sample), and CINT (Spanish sample), specializing in social studies with the use of a computer assisted web interview (CAWI). Informed consent was obtained from all participants. As compensation for their participation, respondents were awarded points that were redeemable for various rewards offered by online panel providers. The study was approved by the Ethics Board of the University of Warsaw.

The study was conducted in 2019 in Spain and Poland. A representative nationwide sample was used in the study in both countries, which reflected the structure of the given country in terms of sex, age, and the size of place of residence. Moreover, the respondents were bread spread users (chocolate hazelnut, jam or peanut butter), and not chocolate hazelnut bread spread rejectors. Thus, the presented sample represents a subset of the representative sample and individuals who do not reject chocolate cream were extracted from the representative sample. The sample size was *n* = 1336, *n* = 675 in Spain and *n* = 661 in Poland. A full profile of the respondents’ profile is presented in Table 1. All the participants gave their consent to taking part in the study after having familiarized themselves with detailed information on the aim and procedure of the study. The completion of the questionnaire took approximately 40 min.

The study was carried out in March 2019 as part of a larger project executed under the EIT Food “OLEOGELS–healthy and sustainable replacement for saturated fat and palm oil in spreads” grant. The choice of the tested food product consisting of chocolate cream, and the choice of the place where the study was conducted (Spain and Poland) was dictated by the purpose of the grant. Only a portion of the results from the consumer study are presented in this article.

### 2.2. Tools and Procedure

#### 2.2.1. Procedure

The research was carried out in two stages. In stage one, various attitudes toward food and consumer behaviors were measured, of which, the measurements of attitudes toward self-perceived knowledge of healthy eating and the health and ecology orientation were used in this article. The tools that were implemented in the study were created for the purpose of the presented research project. Stage two involved an experiment.

#### 2.2.2. Tools

1. Self-perceived knowledge of healthy eating

The scale of paying attention to the health aspect of food (self-perceived knowledge of healthy eating) was based on four statements to which the respondents referred on a scale from 1 to 5 (1-definitely disagree, 5-definitely agree). These statements were:

1. know which products are healthy and which are not;
2. I know the composition of the products I buy;
3. I know the principles of healthy eating;
4. I am looking for information on healthy eating.

For the purpose of the analysis, we used the mean score of the above items. The higher the score on the scale, the greater the importance given to the health dimension of food. The reliability of the scale measured using Cronbach’s alpha was 0.77, which allows the scale to be considered reliable.

2. Health Orientation

The evaluation of eating habits in health terms was measured by asking the question of “How do you evaluate your eating habits? Please choose the statement that suits you best”. The respondents answered on a scale from 1 to 5: 1—I do not eat healthily, 2—I usually do not eat healthily, 3—I eat fairly healthily, 4—I eat rather healthily, 5—I eat healthily (6—I do not care whether I eat healthily or not—this value was not used in the analyses).

3. Ecology Orientation

The ecology orientation was measured by asking a question about purchasing decisions: “I buy mainly natural, ecological products”. The respondents answered this question on a scale from 1—I strongly disagree, to 5—I strongly agree.

4. Experiment

The participants were randomly assigned to one of three experimental conditions. In each condition, the respondents read one of three versions of text describing a new chocolate bread spread entering the market. The basic description was (control condition): “New chocolate hazelnut cream. We present to you a new chocolate hazelnut cream with a velvety smooth, creamy texture and wonderfully rich taste that will delight even the most demanding palate. Great tasting cream with the perfect spreading consistency”. In the second condition (health benefits communication), the title of the description was: “New chocolate hazelnut cream–healthy because its palm oil free”. In the third condition (ecological benefits communication), the title of the description was: “New chocolate hazelnut cream–environmentally friendly because its palm oil free”.

5. Product Evaluation (Dependent Variable)

Afterwards, the respondents were asked to answer the following question: “How do you think, based on this description, what will this new chocolate spread be like?”. The respondents assessed the cream perception based on the description in terms of two aspects: whether it is worth trying and if it is better than the products currently available on the market, and they answered on a scale from 1 to 5 (1—definitely not, 5—definitely yes). Pearson’s correlation between two variables was moderate, 0.56, *p* < 0.001. For the purpose of further analysis, the mean of these two questions was calculated and then used as a single variable product evaluation.

### 2.3. Data Processing and Statistical Analysis

Statistical analyses were performed using IBM SPSS Statistic software, Version 26.0.0.1. To test the interaction effects, we used PROCESS macro v.3.5 by Hayes [16] model 1.

## 3. Results

### 3.1. Spanish Consumers’ Sample

In the first step, we conducted regression analysis to show the effect of the manipulation (the effects of the health benefits communication vs. ecology benefits communication) in the Spanish sample. The experimental conditions were dummy coded with the control condition as the reference value. We controlled for gender (0—men, 1—women), age, and the level of education. We found that only the ecology benefits communication condition was a significant predictor of the evaluation of the proposed product (see Table 2). The evaluation of the proposed product in the ecology condition was higher (*M* = 3.89, *SD* = 0.76) than in the control condition (*M* = 3.71, *SD* = 0.66), but there were no differences between the health condition (*M* = 3.75, *SD* = 0.79) and the control condition. This result partially confirms H1. The controlled demographic variables did not have a significant effect on the evaluation of the product.

In the next step, we wanted to examine the relationship between the evaluation of the product and its individual characteristics (independently from the experimental condition). There was a positive (albeit low) correlation between the evaluation of the described product and the health orientation, *r*(675) = 0.22, *p* < 0.001, as well as the ecology orientation, *r*(675) = 0.107, *p* = 0.005. We failed to observe any significant correlation between the dependent variable and the self-perceived knowledge of healthy eating, *r*(675) = −0.07, *p* = 0.08.

Next, we decided to examine the interaction effect of three experimental conditions and three moderators (health orientation, ecology orientation, and self-perceived knowledge of health eating). To test the interaction effects, we used PROCESS macro v.3.5 by Hayes [16] model 1. There were no significant moderating effects of the health orientation, the ecology orientation, and the self-perceived knowledge of healthy eating for both the health benefits communication and the ecology benefits communication conditions.

The conducted analysis showed that among the whole group of Spaniards, the description of the product referring to its ecological values (ecological condition) influenced the evaluation of the product. The product was seen as worth trying and better than other products already available on the market. As it turns out, for Spanish consumers, references to the ecological value of palm oil free products makes the product more attractive. However, it is worth noting that the manner of evaluating the presented product in terms of whether it was presented as eco-friendly or good for health was not affected by eating style among the Spanish consumers. Regardless of whether the subjects paid attention to the ecological aspects of the food or its healthiness, and if they assessed their diet as healthy or not, they assessed the products in the same way under both conditions.

### 3.2. Polish Consumers’ Sample

For the Polish sample, similarly to the Spanish sample, we performed linear regression analysis to investigate the effects of health benefits vs. ecology benefits communication. The experimental conditions were dummy coded with the control condition as a reference value. We controlled for gender (0—men, 1—women), age, and level of education. Neither the health benefits communication nor the ecology benefits communication conditions were significant predictors of the evaluation of the proposed product (see Table 2). There were no significant differences between the evaluation of the proposed product in the three conditions: ecology (*M* = 3.55, *SD* = 0.74), health (*M* = 3.47, *SD* = 0.74), and control (*M* = 3.47, *SD* = 0.74).

In the next step, we examined the relationship between the product evaluation and the individual characteristics. The evaluation of the product was positively correlated with the health orientation, *r*(661) = 0.29, *p* < 0.001, the ecology orientation, *r*(661) = 0.17, *p* < 0.001, and the self-perceived knowledge of healthy eating; however, this relation was marginally significant, *r*(632) = 0.08, *p* = 0.05.

Next, as in the sample of Spanish consumers, we decided to examine the interaction effect between the three experimental conditions and the three moderators (health orientation, ecology orientation, and self-perceived knowledge of health eating). There was no significant moderating effect of the self-perceived knowledge of healthy eating for both the health benefits communication and the ecology benefits communication conditions compared to the control condition *B* = −0.20, *SE* = 0.10, *p* = 0.05; *B* = −0.04, *SE* = 0.10, *p* = 0.67, respectively.

**Figure 1:**
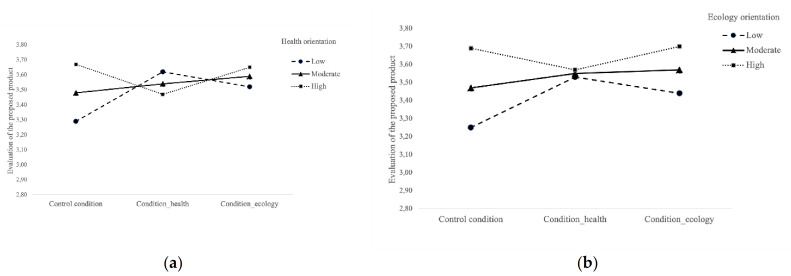
Interaction effect of the experimental condition and of (**a**) health orientation, and (**b**) ecological orientation (Polish consumers sample).

The health orientation significantly moderated the effect of the health benefits communication condition, *B* = −0.33, *SE* = 0.09, *p* < 0.001, showing that communication focused on health (versus the control condition) was successful only for people who evaluated their dietary style as unhealthy (assuming a moderator value of–1 *SD*: *B* = 0.33; *SE* = 0.10; *p* < 0.001; and assuming a moderator value of +1 *SD*: *B* = −0.20; *SE* = 0.10; *p* = 0.05; see Figure 1a). There was no significant moderating effect on the ecology benefits condition vs. the control condition, *B* = −0.15, *SE* = 0.09, *p* = 0.09. The presented results did not confirm H2–contrary to the predictions, communication referring to health benefits (versus the control) had a greater impact on the positive assessment of a product in less health-oriented people.

The ecological orientation also significantly moderated the effect of the health benefits communication condition, *B* = −0.22, *SE* = 0.07, *p* = 0.04, showing that communication focused on health (versus the control) has a significant impact on people who evaluate their ecological attitude as low (assuming a moderator value of–1 *SD*: *B* = 0.28; *SE* = 0.09; *p* < 0.01; and assuming a moderator value of +1 *SD*: *B* = −0.12; *SE* = 0.10; *p* = 0.24; see Figure 1b). Additionally, there was no significant moderating effect on the ecology benefits condition vs. the control condition, *B* = −0.09, *SE* = 0.08, *p* = 0.22.

The conducted analysis showed that an influence of the self-perceived knowledge of healthy eating on the evaluation of the communication relating to palm oil free products can be observed among Poles. Both people who assessed their diet as unhealthy and those who were not environmentally friendly reacted better to the message about the health-related values of the product (healthy chocolate bread spread) and assessed it as worth trying and better than other products available on the market. This suggests that health benefits communication fits better to people who have no prior health and ecology orientation. For those who were not environmentally friendly in their dietary choices (did not buy eco-friendly products), a product with information regarding its health benefits was also rated better.

## 4. Discussion

The conducted study provides some additional insights into the manner of communicating palm oil free products. Our research has shown that in the case of new palm oil alternatives, it is not only how the product is described and the benefits that will be highlighted but also the individual characteristics of the respondent and their nationality that play important roles.

The results of the study partially confirmed hypotheses. First, palm oil free product communication referring to specific environmental benefits was better perceived than communication not referring to any benefits at all or referring to health benefits; however, this was observed only among Spanish consumers. Thus, the second part of the first hypothesis stating that communication referring to health benefits is more effective was not confirmed. It turned out, at least for the Spanish consumers, that communication referring to ecology (not harmful to nature) had a more positive impact on the perception of a product without palm oil than references to health benefits. This result is in line with reports stating that Spaniards are more concerned about ecology in general [17] although it enriches the findings with additional knowledge about the ecological aspects of food. This outcome was not observed among Polish consumers probably because Eastern European countries have a shorter ecological tradition and, although the ecological product market is growing, people are still demonstrating less eco-friendly behavior. This shows that culture plays an important role when it comes to food and its values. People from Spain and Poland not only have a different approach to ecology in general but also respond to communication regarding food values in disparate ways. Another question arises in the context of these findings, namely, why did the health-related benefits remain unconvincing to the Spaniards? Perhaps this was, to some extent, because appealing to health in relation to a product with questionable health benefits (such as chocolate cream) is not an effective strategy after all.

The above findings add valuable knowledge to campaigns aimed to promote palm oil free products or palm oil substitutes, demonstrating that it is important to include the cultural context. Although the demographic variables (not only place of living but also gender and age) were extensively studied in the ecological context, they seem to be less relevant in the context of palm oil. Our data demonstrated that, both in Spain and in Poland, the reaction to palm oil free products was determined more by the type of communication (Spain) and the individual approach to health and ecology in the context of food (Poland) than by demographics. The design of a marketing campaign aiming to convince consumers to buy palm oil free products or products containing palm oil substitutes should also factor in consumer attitudes toward ecology and health in the context of food and the target group should also be carefully defined taking these variables into account.

The results obtained by us can be translated into the creation of marketing campaigns not only in the area of palm oil but also in other fields, such as other environmentally harmful product substitutes. Other managerial implications concern the target group of the product and the communication. It is evident from the foregoing analysis that the perception of product communication can be different depending on the consumer’s features and especially their values. Therefore, a segmentation-based approach to product marketing communication is advised.

One limitation of this research may be that the behaviors measured in the study were based on declarations only and not an actual measurements of consumer behavior. In future research, it would be worth examining the actual behaviors of consumers. Studies show that people declare that they want to eat healthier or that health or ecology are important to them, but this is not always followed by a change in behavior. Despite people declaring being open to healthy products, consumers often do not ultimately choose them [18]. What is more, people’s healthy product choices can be influenced not only by their explicit beliefs but also by their implicit attitudes [19]. This means that consumer food choices are not only affected by what they think about a given product and what they believe in, but also by what remains unconscious to them, and this can still exert an influence on their behavior. Implicit attitudes should also be taken into consideration in future studies regarding the reactions to palm oil free products.

A second limitation of the study is its ecological topic. A pro-ecological orientation can be understood in two ways. Firstly, a person can be environmentally friendly in his or her consumer choices, trying to choose products that do not harm the environment. The second understanding is much more self-centered and concerns opting for ecological products because they are less harmful to consumer health. In this study, the communication of the environmental benefits in the product information referred to fostering the environment. However, the question concerning the pro-ecological orientation referred to an egocentric motivation, namely, the choice of naturally produced goods. In the next study, it would be worthwhile to ensure greater distinction of measurements regarding ecology, and to measure an environmental orientation that is focused on environmental protection.

The third limitation of the study relates to the product being tested. Chocolate cream is a product that is generally criticized from a health perspective (high sugar and fat content). Thus, the appeal in communication to health may also have certain limitations. For this reason, in future studies it would be worthwhile to see just how effective appealing to health vs. the environmental benefits would be for products with explicit health features.

## Figures and Tables

**Table 1:** Respondents’ profile in the Spanish and Polish sample.

Variables	Spanish Consumers (*n* = 675)	Polish Consumers (*n* = 661)
Gender		
Male	49% (*n* = 329)	40% (*n* = 262)
Female	51% (*n* = 346)	60% (*n* = 399)
Age	*M* = 41.26, *SD* = 14.00	*M* = 41.22, *SD* = 14.13
18–24 y.o.	16% (*n* = 107)	15% (*n* = 96)
25–34 y.o.	20% (*n* = 132)	22% (*n* = 145)
35–44 y.o.	22% (*n* = 151)	22% (*n* = 144)
45–54 y.o.	22% (*n* = 151)	21% (*n* = 143)
>55 y.o.	20% (*n* = 134)	20% (*n* = 133)
Education		
Primary	5% (*n* = 32)	3% (*n* = 24)
Vocational	21% (*n* = 141)	13% (*n* = 83)
Secondary	24% (*n* = 163)	45% (*n* = 296)
Higher	50% (*n* = 339)	39% (*n* = 258)
Area of residence		
Rural	11% (*n* = 75)	38% (*n* = 252)
Small urban (up to 20.000)	12% (*n* = 82)	14% (*n* = 89)
Medium urban (up to 99.000)	24% (*n* = 165)	18% (*n* = 120)
Big urban (up to 500.000)	29% (*n* = 193)	18% (*n* = 118)
Large urban (above 500.000)	24% (*n* = 160)	12% (*n* = 82)

**Table 2:** Linear regression analysis predicting the evaluation of the proposed product in Spanish and Polish consumer samples; B = unstandardized beta, SE = standard error.

Variables	Spanish Sample		Polish Sample	
	B	SE	B	SE
Constant	3.86 **	0.14	3.08 **	0.15
Condition_health	0.02	0.03	0.08	0.04
Condition_ecology	0.09*	0.03	0.12	0.07
Gender	0.06	0.06	0.04	0.06
Age	0.003	0.002	0.004	0.002
Education	0.03	0.03	0.06	0.04
R^2^	0.14		0.02	

* *p* < 0.05, ** *p* < 0.001.

## Data Availability

The data presented in this study are openly available in RepODdoi:10.18150/5ARHPO.
